# Prescription for Change: A Web-Based Cross-Sectional Survey on How Physicians’ Relationships with Pharma Reps Are Associated with Views on Stricter Industry Guidelines

**DOI:** 10.31662/jmaj.2025-0103

**Published:** 2026-02-27

**Authors:** Michioki Endo, Hiroaki Saito, Yoshitake Takebayashi, Michio Murakami, Tetsuya Tanimoto, Akihiko Ozaki

**Affiliations:** 1 Department of Surgery, Hyogo Prefectural Awaji Medical Center, Sumoto, Hyogo, Japan; 2 Department of Internal Medicine, Soma Central Hospital, Soma, Fukushima, Japan; 3 Department of Health Risk Communication, Fukushima Medical University School of Medicine, Fukushima, Japan; 4 Center for Infectious Disease Education and Research (CiDER), The University of Osaka, Suita, Osaka, Japan; 5 Department of Internal Medicine, Navitas Clinic Kawasaki, Kanagawa, Japan; 6 Medical Governance Research Institute, Minato, Tokyo, Japan; 7 Breast and Thyroid Center, Jyoban Hospital of Tokiwa Foundation, Iwaki, Fukushima, Japan

**Keywords:** pharmaceutical industry regulations, physician-pharmaceutical relationships, ethical guidelines, medical representatives, Japanese physicians

## Abstract

**Introduction:**

Japan, the world’s third-largest pharmaceutical market, introduced new guidelines in 2019 to address ethical concerns in pharmaceutical company-physician relationships. This study aimed to analyze Japanese physicians’ attitudes toward these strengthened regulations.

**Methods:**

An online survey of 1,203 Japanese physicians was conducted in November 2019. Respondents were categorized based on their frequency of interaction with pharmaceutical companies: frequent (82, 6.8%), moderate (930, 77.3%), or rare (191, 15.9%). The survey assessed awareness of the new guidelines, perceived changes in promotional activities, and attitudes toward stricter regulations. Multivariable modified Poisson regression was used to identify factors associated with opposition to the regulations.

**Results:**

A total of 640 (53.3%) respondents opposed stricter regulations, while 325 (27.1%) were in favor. Physicians with frequent (adjusted Incidence Rate Ratio [aIRR] 1.61, 95% confidence interval [CI] 1.24-2.10) or moderate (aIRR 1.53, 95% CI 1.24-1.89) interactions were more likely to oppose regulations compared with those with rare interactions. Hospital directors/managers, those affiliated with private institutions, and recent graduates also showed higher opposition. The main reason for favoring regulations was to promote healthy industry relationships (38, 9.3%), while the primary concern among those opposed was related to information gathering or potential patient disadvantages (88, 21.6%).

**Conclusions:**

The study reveals diverse views on industry-physician relations in Japan. Frequent interactions and leadership roles correlate with opposition to stricter regulations. The results suggest a need for balanced policies that consider varied perspectives.

## Introduction

The financial relationships between pharmaceutical companies and healthcare professionals not only have the potential to create conflicts of interest in healthcare ^(1), (2)^, but can also be the source of various scandals ^(3), (4), (5)^. Among the wide-ranging efforts to address this issue, the most concrete and practical measure is improving transparency in these financial relationships. In many countries, details of honoraria and donations to healthcare professionals and institutions are disclosed either through legal obligations or voluntary initiatives by pharmaceutical companies ^(6), (7), (8), (9)^. Based on this information, detailed studies on the magnitude and distribution of funds paid are being conducted ^(1), (10), (11), (12)^. Furthermore, regulations restricting promotional activities of pharmaceutical companies have been adopted as an effective measure to some extent ^(13)^. Specifically, industry organizations such as the International Federation of Pharmaceutical Manufacturers & Associations and the European Federation of Pharmaceutical Industries and Associations have been developing and revising guidelines (GLs) on pharmaceutical companies’ promotional activities, achieving notable results in this area ^(14), (15)^.

Japan has the third-largest pharmaceutical market in the world. According to IQVIA, in 2023, Japan’s pharmaceutical market grew 3.1% to ¥11,280,631 million (US$102.5 billion), exceeding ¥11 trillion (US$100.0 billion) for the first time ^(16)^. Financial relationships between pharmaceutical companies and healthcare professionals have caused multiple scandals, including the Diovan incident ^(3), (4), (5)^. In response, GLs were introduced to increase transparency in these relationships. Since 2013, under these GLs, details of honoraria and donations to healthcare professionals have been disclosed by pharmaceutical companies ^(17)^. Furthermore, in April 2019, the Guidelines for Provision of Sales Information for Prescription Drugs was announced by the Ministry of Health, Labour and Welfare ^(18)^.

The pharmaceutical industry has since witnessed the implementation of new GLs imposing stricter regulations on information provision and the offering of benefits by pharmaceutical companies ^(18)^. Consequently, these companies have generally become more restrained in providing excessive benefits, as exemplified by the cessation of condolence money offerings and the prohibition of product names on stationery. Additionally, most pharmaceutical companies have voluntarily limited the dissemination of information about unapproved drugs and abstained from making comparative claims against competitors’ products. Similar constraints have also been extended to company-sponsored lectures. While these actions should be considered a welcome development in terms of ethical standards, the resulting self-imposed restrictions have unintentionally raised concerns among healthcare professionals who rely on such benefits and information in clinical settings. It is noteworthy, however, that this particular concern regarding potential impacts on clinical practice has not yet been subject to rigorous empirical investigation.

The authors previously conducted an online survey and found that physicians who frequently interact with pharmaceutical companies tend to make prescribing decisions based on information obtained from these companies ^(19)^. A meta-analysis revealed that physicians who had interactions with pharmaceutical companies, including but not limited to representatives, were more than twice as likely to prescribe promoted drugs inappropriately or demonstrate lower prescribing quality compared with physicians who did not have such interactions ^(20)^. This association extended beyond interactions with sales representatives to include various forms of pharmaceutical company influence, such as receiving gifts, attending sponsored educational events, and other types of industry involvement ^(20)^. Consequently, we formulated the hypothesis that healthcare professionals who frequently interact with pharmaceutical companies are likely to be skeptical of new regulations restricting these interactions.

This study aimed to analyze in detail the relationship between awareness of and attitudes toward the Guidelines for Provision of Sales Information for Prescription Drugs issued in Japan in April 2019. Specifically, it sought to clarify how the frequency of interaction with pharmaceutical companies influences these attitudes. Additionally, it aimed to summarize the actual opinions of healthcare professionals regarding this new GL.

## Materials and Methods

### Setting and participants

The sampling methodology for this online survey is detailed elsewhere ^(19)^. Conducted from November 1 to 12, 2019, in collaboration with M3, Inc., Japan’s largest medical staffing company, the survey targeted Japanese physicians registered on m3.com. This platform, which claims over 90% of Japanese physicians as members, offers various healthcare services. To achieve a 95% confidence level with a 5% margin of error, we aimed for approximately 1,200 respondents. The sample was designed to match national statistics in age distribution (above/below 40 years) and workplace type (hospital/clinic). Other variables were addressed by random invitation to reduce potential confounding factors.

While the initial analysis aimed to extract factors associated with physicians who frequently reference information obtained from pharmaceutical company medical representatives (MRs) or company-sponsored seminars, using both indirect and direct methods, the current study utilized the same dataset to analyze different aspects. This study focused on awareness, attitudes, and opinions regarding the Guidelines for Provision of Sales Information for Prescription Drugs established in 2019. Specifically, it examined how the frequency of interactions with pharmaceutical companies influences agreement or disagreement with this GL and sought to summarize the range of opinions expressed about it.

In our previous study, we excluded the fastest 10% of respondents to eliminate potentially inaccurate responses and conducted our analysis on the remaining sample. For this current study, however, one of our aims was to capture a diverse range of opinions about the GL. Consequently, we deliberately chose not to exclude this 10%. Nevertheless, we also conducted a sensitivity analysis excluding the fastest 10% of respondents to compare results.

#### Development of the survey instrument

The survey instrument was developed by referencing previous research while considering the specific context of the Japanese healthcare industry ^(21), (22)^. The questionnaire consisted of 25 items and underwent a review process involving two physicians (HS, AO) and two researchers (MM, YT). Prior to the full-scale implementation of the survey, the questionnaire was subjected to a preliminary review by a research team member who was not directly involved in the study’s development. This review focused on assessing the readability, clarity, and comprehensibility of the questions. A comprehensive list of the survey questions is available in the previously published study ^(19)^.

### Independent variables

#### Basic characteristics

From the obtained database, we used data on the basic characteristics of clinicians, including gender (male or female); age (under 40 or 40 and above); graduation year from medical school; specialty (Internal Medicine, Respiratory Medicine, Gastroenterology, Cardiology, Pediatrics, Psychiatry, Psychosomatic Medicine, Allergy, Rheumatology, Surgery, Orthopedic Surgery, Plastic Surgery, Cosmetic Surgery, Neurosurgery, Thoracic Surgery, Cardiovascular Surgery, Obstetrics and Gynecology, Obstetrics, Gynecology, Ophthalmology, Otolaryngology, Dermatology, Urology, Rehabilitation Medicine, Radiology, Anesthesiology, and Others); type of affiliation (national/public university hospital, private university hospital, national/public hospital, private hospital, national/public clinic, private clinic, or other); position (hospital director/management, department head/professor, no specific position, or other); prefecture of work; and work status (full-time, full-time plus part-time, or part-time only).

### Interactions with pharmaceutical companies

We also used data on interaction with pharmaceutical companies, including the duration of a single meeting with MRs (10 minutes or less, 11-20 minutes, 21-30 minutes, 31-60 minutes, or 61 minutes or more) and the frequency of these meetings (daily, 2-3 times per week, once a week, twice a month, less than that, or never).

Additionally, we collected information on the frequency of attending in-hospital briefings sponsored by pharmaceutical companies (daily, 2-3 times per week, once a week, twice a month, less than that, or never) and the frequency of participating in off-site study sessions, training sessions, or lectures sponsored by pharmaceutical companies (daily, 2-3 times per week, once a week, twice a month, less than that, or never). As previously explained ^(19)^, we created a composite variable for the frequency of interaction with MRs based on these three survey items. Respondents were categorized into three groups: “Frequent interaction” (all items at least weekly), “Rare interaction” (all items less than monthly or never), and “Moderate interaction” (all other combinations).

### Awareness of the GLs for the provision of sales information for prescription drugs and perceived changes following implementation

We also extracted data on awareness of the Guidelines for Provision of Sales Information for Prescription Drugs established in 2019 and perceptions of changes in the activities of pharmaceutical companies following the GLs’ implementation. Participants were asked about their familiarity with the GLs. The survey further explored respondents’ observations of changes in pharmaceutical MRs’ behavior, such as alterations in the frequency of various interactions (e.g., meetings with MRs, in-hospital briefings, and out-of-hospital educational events) and the depth of information provided.

### Dependent variable

#### Attitudes toward the GLs for provision of sales information for prescription drugs

Attitudes toward the increased regulation of pharmaceutical companies’ information provision activities were assessed using a 5-point Likert scale, allowing respondents to express their level of agreement or disagreement with the stricter oversight.

### Data analysis

We first conducted descriptive analyses on the participating physicians’ basic characteristics, their interactions with pharmaceutical companies, and their responses to questions related to the GL implemented in 2019. We excluded the frequency of interactions with pharmaceutical companies and their component variables from the characteristics analyzed in this study, as these variables were used for participant stratification.

To examine the relationship between the frequency of interactions with pharmaceutical companies and attitudes toward stricter regulations, we constructed a modified Poisson regression model with robust standard errors. For this model, we dichotomized responses to the dependent variable “Attitudes toward the Guidelines for Provision of Sales Information for Prescription Drugs”: “Agree,” “Somewhat agree,” and “Neutral” were grouped together, while “Disagree” and “Somewhat disagree” formed the other group. We included all independent variables as covariates, including basic characteristics, interactions with pharmaceutical companies, awareness of the GLs for provision of sales information for prescription drugs, and perceived changes following implementation. The frequency of interaction with pharmaceutical companies served as our primary variable of interest.

We employed backward elimination for variable selection. The threshold for statistical significance was set at *p* ≤ 0.05. All data analyses were performed using Stata MP 14.0 (Stata Corp, TX, United States).

In this study, we solicited free comments from participants and analyzed them using the following procedure. First, OA, HS, and YT independently reviewed the free comments multiple times. Initially, we intended to create question categories based on whether respondents agreed, were neutral, or disagreed with the GLs restricting interactions with pharmaceutical companies, seeking detailed explanations in the free comments. However, our review revealed that even among those who generally agreed with the GLs, there were comments expressing opposition, and vice versa.

Consequently, we revised our approach and established the following common categories for all comments:

1. Agreement (comments focusing on money and collusion, e.g., “Previous practices were excessive,” “A healthier relationship is desirable”)

2. Agreement (comments mentioning etiquette, burden, or comfort, e.g., “It reduces burden,” “It’s more comfortable,” “MRs are impolite”)

3. Agreement (comments focusing on patient benefits, e.g., “It’s beneficial for patients,” “It leads to reduced drug prices”)

4. Agreement (comments focusing on alternative information sources, e.g., “Information can be gathered online,” “There are other methods,” “MRs are unnecessary”)

5. Disagreement (comments tolerating financial transactions or gift-giving with pharmaceutical companies, e.g., “It’s fine within reasonable limits,” “The current situation is acceptable”)

6. Disagreement (comments focusing on information gathering or patient disadvantages, e.g., “Sufficient information isn’t provided,” “MRs are necessary”)

7. Comments that were difficult to categorize or where agreement or disagreement was unclear

Of the 1,203 respondents, 408 (33.9%) provided meaningful written comments. Each researcher independently classified these comments according to the established categories. We then calculated Kappa statistics to assess inter-rater reliability between pairs of researchers: OA and HS (κ = 0.53, 66.7% agreement), HS and YT (κ = 0.49, 65.9% agreement), and YT and OA (κ = 0.70, 78.2% agreement). These values were deemed acceptable. All three researchers agreed on the classification for 237 (58.1%) of the 408 comments. For the remaining 171 (41.9%) comments where consensus was not initially reached, we held a meeting to finalize their categorization. We present the proportion of responses in each category based on this final classification.

### Informed consent and ethical approval

The questionnaire was distributed with a description of the study. Informed consent was considered given upon questionnaire completion. The Ethics Committee of the Medical Governance Research Institute (MG2019-05-R3) and Fukushima Medical University (2019-326) reviewed and approved this study.

## Results

The total number of participants was 1,203, of whom 82 (6.8%) were in the frequent interaction group, 930 (77.3%) in the moderate interaction group, and 191 (15.9%) in the rare interaction group.

Table 1 shows the sociodemographic characteristics of the participants. Females accounted for 150 participants, representing 12.5% of the total, and those 40 years old or older numbered 606, accounting for 50.4% of the total. Regarding graduation year, those who graduated in the 2000s were the most numerous at 361, representing 30.0% of the total. Among affiliations, those working at private hospitals were the most common at 369 (30.7%). Regarding job titles, 628 participants (52.2%) held no specific title. For work status, 899 (74.7%) were full-time employees. In terms of departments, internal medicine physicians were the most common at 280 (23.3%). Concerning the duration of individual meetings with MRs, the most common duration was 10 minutes or less for 756 participants (66.8%).

**Table 1.** Characteristics of Respondents.

Variable	Total (n = 1203)	Frequent interaction (n = 82)	Moderate interaction (n = 930)	Rare interaction (n = 191)
Female, n (%)	150 (12.5)	11 (13.4)	90 (9.7)	49 (25.7)
Age, n (%)				
< 40 years old	597 (49.6)	49 (59.8)	455 (48.9)	93 (48.7)
≥40 years old	606 (50.4)	33 (40.2)	475 (51.1)	98 (51.3)
Graduate year				
1950s or before	16 (1.3)	2 (2.4)	13 (1.4)	1 (0.5)
1960s	22 (1.8)	1 (1.2)	17 (1.8)	4 (2.1)
1970s	67 (5.6)	3 (3.7)	52 (6.0)	12 (6.3)
1980s	243 (20.2)	13 (15.9)	203 (21.8)	27 (14.1)
1990s	194 (16.1)	9 (11.0)	145 (15.6)	40 (20.9)
2000s	361 (30.0)	26 (31.7)	266 (28.6)	69 (36.1)
2010s	300 (24.9)	28 (34.2)	234 (25.2)	38 (19.9)
Type of affiliations, n (%)				
National/public university hospital	212 (17.6)	23 (28.1)	162 (17.4)	27 (14.1)
Private university hospital	77 (6.4)	13 (15.9)	59 (6.3)	5 (2.6)
National/public hospital	247 (20.5)	13 (15.9)	201 (21.6)	33 (17.3)
Private hospital	369 (30.7)	22 (26.8)	277 (29.8)	70 (36.7)
National/public clinic	17 (1.4)	1 (1.2)	14 (1.5)	2 (1.1)
Private clinic	268 (22.3)	10 (12.2)	214 (23.0)	44 (23.0)
Other	13 (1.1)	0 (0.0)	3 (0.3)	10 (5.2)
Position, n (%)				
Hospital director/manager	109 (9.1)	9 (11.0)	92 (9.9)	8 (4.2)
Clinic director	141 (11.7)	8 (9.8)	128 (13.8)	5 (2.6)
Department head/professor	266 (22.1)	18 (22.0)	213 (22.9)	35 (18.3)
No specific position	628 (52.2)	43 (52.4)	456 (49.0)	129 (67.5)
Other	59 (4.9)	4 (4.9)	41 (4.4)	14 (7.3)
Work status, n (%)				
Full-time	899 (74.7)	62 (75.6)	719 (77.3)	118 (61.8)
Full-time + part-time	196 (16.3)	12 (14.6)	151 (16.2)	33 (17.3)
Part-time only	108 (9.0)	8 (9.8)	60 (6.5)	40 (20.9)
Specialty, n (%)				
Internal Medicine	280 (23.3)	31 (37.8)	212 (22.8)	37 (19.4)
Respiratory Medicine	40 (3.3)	5 (6.1)	33 (3.6)	2 (1.1)
Gastroenterology	89 (7.4)	5 (6.1)	77 (8.3)	7 (3.7)
Cardiology	76 (6.3)	8 (9.8)	56 (6.0)	12 (6.3)
Pediatrics	92 (7.7)	1 (1.2)	79 (8.5)	12 (6.3)
Psychiatry	96 (8.0)	5 (6.1)	75 (8.1)	16 (8.4)
Psychosomatic Medicine	3 (0.3)	0 (0.0)	1 (0.1)	2 (1.1)
Allergology	1 (0.1)	0 (0.0)	1 (0.1)	0 (0.0)
Rheumatology	12 (1.0)	2 (2.4)	10 (1.1)	0 (0.0)
Surgery	67 (5.6)	3 (3.7)	56 (6.0)	8 (4.2)
Orthopedics	67 (5.6)	5 (6.1)	57 (6.1)	5 (2.6)
Plastic Surgery	11 (0.9)	0 (0.0)	5 (0.5)	6 (3.1)
Neurosurgery	25 (2.1)	1 (1.2)	22 (2.4)	2 (1.1)
Thoracic Surgery	7 (0.6)	0 (0.0)	3 (0.3)	4 (2.1)
Cardiovascular Surgery	11 (0.9)	0 (0.0)	8 (0.9)	3 (1.6)
Obstetrics and Gynecology	34 (2.8)	2 (2.4)	21 (2.3)	11 (5.8)
Obstetrics	3 (0.3)	0 (0.0)	3 (0.3)	0 (0.0)
Gynecology	3 (0.3)	0 (0.0)	1 (0.1)	2 (1.1)
Ophthalmology	24 (2.0)	2 (2.4)	19 (2.0)	3 (1.6)
Otolaryngology	35 (2.9)	0 (0.0)	31 (3.3)	4 (2.1)
Dermatology	41 (3.4)	4 (4.9)	33 (3.6)	4 (2.1)
Urology	21 (1.8)	0 (0.0)	19 (2.0)	2 (1.1)
Rehabilitation Medicine	8 (0.7)	0 (0.0)	4 (0.4)	4 (2.1)
Radiology	34 (2.8)	0 (0.0)	28 (3.0)	6 (3.1)
Anesthesiology	47 (3.9)	4 (4.9)	24 (2.6)	19 (10.0)
Other	76 (6.3)	4 (4.9)	52 (5.6)	20 (10.5)
Duration of individual meetings with MRs, n (%)				
10 minutes or less	756 (66.8)	47 (57.3)	609 (66.7)	100 (73.5)
11-20 minutes	283 (25.0)	29 (35.4)	230 (25.2)	24 (17.7)
21-30 minutes	73 (6.5)	4 (4.9)	61 (6.7)	8 (5.9)
31-60 minutes	19 (1.7)	2 (2.4)	13 (1.4)	4 (2.9)

MR: medical representative.

The “rare interaction” group had a higher proportion of women at 25.7% (49/191) compared with the “frequent interaction” group (13.4%, 11/82) and the “moderate interaction” group (9.7%, 90/930). In contrast, the “frequent interaction” group tended to include more younger participants, with 59.8% (49/82) under 40 years old, compared with 48.9% (455/930) in the moderate group and 48.7% (93/191) in the rare group. Additionally, the “frequent interaction” group had a higher proportion of individuals affiliated with universities (44.0%, 36/82), while the proportion of those in private clinics was lower (12.2%, 10/82). From the perspective of job titles, the “rare interaction” group had a higher proportion of those with “no job title” (67.5%, 129/191) and a higher rate of “part-time employment” (20.9%, 40/191). Furthermore, from the department perspective, the “frequent interaction” group had a particularly high proportion of “Internal Medicine” practitioners (37.8%, 31/82), which differed from the overall trend (23.3%, 280/1203).

Table 2 summarizes the understanding, implementation, and attitudes toward the newly established GLs according to the frequency of meetings with MRs. A total of 240 participants (20.0%) were aware of and understood the new GLs’ contents, while 694 participants (57.7%) were aware that new GLs had been published but did not understand their contents. This exceeded the proportion of those who understood the contents. Meanwhile, 269 participants (22.4%) were not aware of the publication of the GLs.

**Table 2.** The Impact of Medical Representative Interaction Frequency on Physicians’ Guideline Understanding, Implementation, and Perspectives.

Variable	Total (n = 1203)	Frequent interaction (n = 82)	Moderate interaction (n = 930)	Rare interaction (n = 191)
Awareness and understanding about the GL, n (%)				
Be aware of the GL publication and understand its contents	240 (20.0)	31 (37.8)	194 (20.9)	15 (7.9)
Be aware of the publication of the GL but not understand its contents	694 (57.7)	42 (51.2)	539 (58.0)	113 (59.2)
Be unaware of the publication of the GL	269 (22.4)	9 (11.0)	197 (21.2)	63 (33.0)
Changes after GL compliance, n (%)				
Decreased frequency of visits with MR	426 (35.4)	38 (46.3)	334 (35.9)	54 (28.3)
Decreased frequency of in-hospital briefings	400 (33.3)	42 (51.2)	312 (33.6)	46 (24.1)
Decreased frequency of out-of-hospital events	426 (35.4)	33 (40.2)	351 (37.7)	42 (22.0)
Content of the information provided by the MR became more superficial than the past	311 (25.9)	26 (31.7)	259 (27.9)	26 (13.6)
Publications of Guidelines and other regulations, n (%)				
Agree	79 (6.6)	9 (11.0)	52 (5.6)	18 (9.4)
Partly agree	246 (20.5)	14 (17.1)	180 (19.4)	52 (27.2)
Neutral	238 (19.8)	8 (9.7)	169 (18.2)	61 (31.9)
Partly disagree	341 (28.4)	23 (28.1)	285 (30.7)	33 (17.3)
Disagree	299 (24.9)	28 (34.2)	244 (26.2)	27 (14.1)

GL: guideline; MR: medical representative.

Regarding changes after GL compliance, 426 respondents (35.4%) felt that MR visits had decreased, 400 (33.3%) noticed a decrease in in-hospital study sessions, 426 (35.4%) noticed a decrease in out-of-hospital lectures, and 311 (25.9%) felt that the information provided by pharmaceutical companies had become more superficial. Concerning the regulations imposed by the GLs on pharmaceutical companies, 341 people (28.4%) partly disagreed, and 299 (24.9%) disagreed, with over half of the respondents (53.3%) opposing the regulations.

Moreover, compared with the overall proportion, those with frequent interactions tended to have a better understanding of the GL contents (37.8%, 31/82 vs. 20.0% overall). They were also more likely to feel that MR visits (46.3%, 38/82 vs. 35.4% overall) and both in-hospital (51.2%, 42/82 vs. 33.3% overall) and out-of-hospital study sessions (40.2%, 33/82 vs. 35.4% overall) had decreased, and that the information provided by pharmaceutical companies had become more superficial (31.7%, 26/82 vs. 25.9% overall). Furthermore, those with frequent interactions tended to be more opposed to the regulations set by the GLs, with 62.3% (51/82) disagreeing or partly disagreeing, compared with 56.9% (529/930) in the moderate interaction group and 31.4% (60/191) in the rare interaction group.

Table 3 shows a multivariable modified Poisson regression model identifying characteristics associated with those who oppose stronger regulations. Physicians with frequent interactions (Adjusted Incidence Rate Ratio (aIRR) 1.61, 95% confidence interval [CI] 1.24-2.10, p < 0.001) and moderate interactions (aIRR 1.53, 95% CI 1.24-1.89, p < 0.001) were more likely to oppose regulations compared to those with rare interactions. Additionally, hospital directors, hospital managers, or clinic directors (aIRR 1.25, 95% CI 1.07-1.46, p = 0.006) were more likely to oppose regulations compared with those with no specific position. Heads of departments/professors also showed a trend toward opposition, though this was not statistically significant (aIRR 1.16, 95% CI 0.997-1.34, p = 0.055).

**Table 3.** Multivariable Modified Poisson Regression Models of Factors Influencing Opposition to Guidelines for Provision of Sales Information for Prescription Drugs.

Variables	aIRR (95%CI)	p-Values
Interaction with MRs		
Rare interaction	1	
Moderate interaction	1.53 (1.24-1.89)	<0.001
Frequent interaction	1.61 (1.24-2.10)	<0.001
Graduate year		
2010s	1	
2000s	0.85 (0.73-0.98)	0.024
1990s	0.69 (0.56-0.84)	<0.001
1980s or before	0.84 (0.71-0.99)	0.035
Types of affiliation		
National and public medical institutions	1	
Private and other medical institutions	1.16 (1.03-1.30)	0.013
Position		
No specific position	1	
Hospital director, hospital manager or clinic director	1.25 (1.07-1.46)	0.006
Head of Department/Professor	1.16 (0.997-1.34)	0.055
Decreased frequency of out-of-hospital events		
No	1	
Yes	1.36 (1.23-1.50)	<0.001
The content of the information provided by the MR became more superficial than in the past		
No	1	
Yes	1.49 (1.35-1.64)	<0.001

CI: confidence interval; aIRR: adjusted incidence rate ratio; MR: medical representative.

Those affiliated with private and other medical institutions (aIRR 1.16, 95% CI 1.03-1.30, p = 0.013) were more likely to oppose regulations compared with those affiliated with national and public medical institutions. Regarding graduation year, those who graduated in the 2000s (aIRR 0.85, 95% CI 0.73-0.98, p = 0.024), 1990s (aIRR 0.69, 95% CI 0.56-0.84, p < 0.001), and 1980s or before (aIRR 0.84, 95% CI 0.71-0.99, p = 0.035) were less likely to oppose regulations compared to those who graduated in the 2010s. Respondents who perceived a decrease in the frequency of out-of-hospital events (aIRR 1.36, 95% CI 1.23-1.50, p < 0.001) and those who felt that the content of information provided by MRs had become more superficial (aIRR 1.49, 95% CI 1.35-1.64, p < 0.001) were also more likely to oppose the regulations. Since there were more women in the rare interaction group (25.7%) compared with the moderate (9.7%) and frequent interaction groups (13.4%), we conducted a stratified analysis by sex, but the results did not change significantly (Supplementary Materials 1 and 2).

Table 4 presents an analysis of healthcare professionals’ comments on the Guidelines for Provision of Sales Information for Prescription Drugs. Among the 408 respondents who provided meaningful comments, several themes emerged. Agreement themes included focusing on money and collusion (38, 9.3% of all comments), etiquette and burden (5, 1.2%), patient benefits (5, 1.2%), and alternative information sources (12, 2.9%). These themes were more prevalent among those who agreed with the regulations. Disagreement themes included tolerating financial transactions or gift-giving (90, 22.1%) and concerns about information gathering or patient disadvantages (88, 21.6%), which were more common among those who opposed the regulations. A significant portion of comments (170, 41.7%) were difficult to categorize.

**Table 4.** Analysis of Healthcare Professionals’ Comments on Guidelines for Provision of Sales Information for Prescription Drugs.

Variables	Total	Agree	Neutral	Disagree
Agreement				
Focusing on money and collusion	38 (9.3)	30 (30.0)	2 (3.5)	6 (2.4)
Focusing on etiquette, burden, or discomfort	5 (1.2)	3 (3.0)	2 (3.5)	0 (0.0)
Focusing on patient benefits	5 (1.2)	4 (4.0)	1 (1.7)	0 (0.0)
Focusing on alternative information sources	12 (2.9)	5 (5.0)	2 (3.5)	5 (2.0)
Disagreement				
Tolerating financial transaction or gift-giving	90 (22.1)	8 (8.0)	4 (6.9)	78 (31.2)
Focusing on information gathering or patient disadvantages	88 (21.6)	7 (7.0)	9 (15.5)	72 (28.8)
Comments difficult to be categorized	170 (41.7)	43 (43.0)	38 (65.5)	89 (35.6)
Missing	795	225	180	390

Supplementary Materials 3-6 present analyses excluding the fastest 10% of respondents to complete the survey. When compared with Table 1, 2, 3 and Table 4, there were no substantial differences in the overall patterns or main findings. The distribution of respondent characteristics, awareness, and understanding of the GLs, perceived changes after GL implementation, and attitudes toward the regulations remained largely consistent. The multivariable modified Poisson regression model in Supplementary Material 5 showed similar associations between various factors and opposition to the GLs as in Table 3, with only minor variations in the magnitude of the adjusted incidence rate ratios. The analysis of healthcare professionals’ comments in Supplementary Material 6 also yielded comparable themes and proportions to those found in Table 4. These similarities suggest that the exclusion of the fastest 10% of respondents did not significantly impact the study’s main findings or conclusions.

## Discussion

This online survey demonstrated that individuals with more frequent interactions with pharmaceutical companies exhibit a stronger tendency to oppose the Guidelines for Provision of Sales Information for Prescription Drugs implemented in 2019, which imposed stricter regulations on information provision and other interactions between pharmaceutical companies and healthcare professionals. Overall, the proportion of those opposing the GLs exceeded 50%. Opponents cited concerns not only about the restriction of simple benefits but also about the potential limitation of necessary information provision for patients. Despite acknowledging the need for such restrictions, the survey results generally indicated a demand for alternative methods of information dissemination.

Consistent with the original hypothesis, the survey indicated that individuals with more frequent interactions with pharmaceutical companies were more inclined to oppose regulations. This tendency for deeper involvement with pharmaceutical companies to correlate with more favorable attitudes toward them is a trend repeatedly observed in previous research ^(2), (19), (20), (23)^, and our survey results align with these past findings. Considering the free-response results, the reasons can be broadly categorized into two main areas. The first is the concern that information obtained from meetings with MRs will become superficial. As a result of the new GLs, pharmaceutical companies have strictly limited the provision of information that might potentially conflict with the GLs’ stipulations, which may be contributing to dissatisfaction among healthcare professionals. The second reason is the loss of tangible benefits. Historically, physicians have benefited from meals, stationery, travel expenses, and similar provisions through MRs. Opposition to the loss of such benefits is likely to be more pronounced among those who have more frequent interactions, as they stand to lose more from these restrictions.

Other factors related to stronger opposition to regulations can be interpreted from a similar perspective. For instance, those in leadership positions demonstrated stronger opposition, likely due to their historically stronger ties with pharmaceutical companies. Additionally, opposition was more pronounced in private hospitals and similar institutions, as such facilities generally have fewer regulations governing interactions with for-profit companies compared with public institutions.

However, the stronger opposition among younger professionals is somewhat counterintuitive. This might be attributed to a growing awareness among younger healthcare professionals about the negative impacts of financial relationships with pharmaceutical companies. Their opposition could reflect a desire for clearer boundaries and more transparent interactions, rather than a wish to maintain the status quo.

Residents, who are younger physicians in training, may exhibit a stronger tendency. However, specific data on this subgroup were not available, and thus detailed analysis could not be performed.

### Clinical implications and perspective

This survey synthesizes field opinions on the pharmaceutical industry’s current regulatory system, highlighting widespread dissatisfaction among clinical physicians. Implemented top-down, the system seems largely detached from healthcare professionals’ day-to-day realities. While it is essential to recognize that recurrent pharmaceutical company misconduct necessitated the establishment of such a regulatory framework ^(3), (4), (5)^, most practicing physicians are not directly involved in these scandals. Various evidence suggests that relationships with pharmaceutical companies should be limited as much as possible ^(20), (23)^. However, moving forward, measures that incorporate more input from healthcare professionals on the front lines should be implemented.

Nevertheless, regardless of whether such measures progress, it is imperative for healthcare professionals to obtain medical information by independently comparing various sources, rather than relying solely on pharmaceutical companies. For pharmaceutical company MRs, the range of information they can provide is becoming increasingly limited. Consequently, it can be argued that the act of providing information itself is of primary importance rather than the specific nature of the information provided. This is because, as revealed in our previous survey ^(19)^, the mere fact of meeting can potentially lead to increased prescriptions. In recent years, there have also been concerns that newly created departments, such as Medical Affairs, are providing scientific information while simultaneously attempting to establish various relationships similar to those that existed with MRs previously ^(24)^.

In light of this, it is encouraging that, according to the free comments in this survey, an increasing number of healthcare professionals are already attempting to acquire necessary medical information independently. Going forward, it is crucial to support and facilitate this trend further. Even in situations where time constraints exist, reliance on information provided by pharmaceutical companies should be minimized to the greatest extent possible.

### Limitations

This survey has three primary limitations. First, its representativeness, previously discussed in detail ^(19)^, warrants consideration. In brief, while hospital employment, university affiliation, and specialty distribution largely aligned with national data, our sample underrepresented female physicians (12.5% vs. 22.8% nationally) and overrepresented those under 40 (46.9% vs. 30.2%). As m3.com users, participants may interact more frequently with pharmaceutical companies than average. Nevertheless, given m3.com’s broad reach (>80% of Japanese physicians) and our substantial sample size, the study provides valuable insights into physician-industry relationships in Japan.

Second, the online nature of the survey may introduce inherent bias, potentially skewing the respondent pool toward more internet-savvy physicians. Third, this study was conducted prior to the coronavirus disease 2019 pandemic, during a time when face-to-face interactions were the norm, and therefore does not fully reflect the changes that have occurred since the pandemic, such as the increase in web-based lectures. As a result, the current situation may differ from the one captured in this study.

Since the pandemic, online interactions such as web-based lectures have become more common, lowering the barrier to participation. There have also been reports indicating that prescribing behaviors have been influenced even after this shift to online formats ^(25)^. In light of these changes, while access to information has become easier, it is more important than ever to critically assess the accuracy and reliability of that information.

### Conclusions

This study elucidated attitudes toward stronger regulations in the relationship between Japanese physicians and pharmaceutical companies. It demonstrated that physicians with more frequent interactions with pharmaceutical companies, those holding specific positions or affiliations, and more recent graduates exhibit a stronger tendency to oppose increased regulations. These results reflect the complex perspectives on the relationship between the pharmaceutical industry and healthcare professionals, indicating the existence of diverse attitudes toward regulatory strengthening. In future policy formulation, it is essential to consider these diverse opinions and strike an appropriate balance of regulation while maintaining the quality of information dissemination. Continuous evaluation and research on the effectiveness and long-term impact of regulations are also imperative.

## Article Information

### Acknowledgments

We thank M3, Inc. for their collaboration in conducting this online survey and all participating physicians for their valuable input.

### Author Contributions

Writing - original draft, review & editing: Michioki Endo. HS: Writing - review & editing: Hiroaki Saito. Writing -review & editing: Yoshitake Takebayashi. Writing -review & editing: Michio Murakami. Writing - review & editing: Tetsuya Tanimoto. Conceptualization, Writing - original draft, review & editing: Akihiko Ozaki.

### Conflicts of Interest

Dr. Saito received personal fees from Taiho Pharmaceutical Co., Ltd outside the scope of the submitted work. Dr. Ozaki received personal fees from MNES, Kyowa Kirin Inc., Becton, Dickinson and Company, and Taiho Pharmaceutical Co., Ltd., outside the scope of the submitted work. Regarding non-financial conflicts of interest among the study authors, Dr. Saito and Ozaki are engaged in ongoing research examining financial and non-financial conflicts of interest among healthcare professionals and pharmaceutical companies in Japan. Dr. Tanimoto receives personal fees from Medical Network Systems, MNES Inc., and Bionics Inc., outside the submitted work. The other authors have no conflicts of interest to disclose.

### Ethics Approval

The Ethics Committee of the Medical Governance Research Institute (MG2019-05-R3) and Fukushima Medical University (2019-326) reviewed and approved this study.

## Supplement

Supplementary Material
