# Supplementary material for: Prescription for Change: A Web-Based Cross-Sectional Survey on How Physicians’ Relationships with Pharma Reps Are Associated with Views on Stricter Industry Guidelines
Source: JMA J. 2026 Feb 27;9(2):457–66. doi: 10.31662/jmaj.2025-0103 (PMC13058698; doi:10.31662/jmaj.2025-0103)
Supplement: Supplementary Material [file 2433-3298-9-2_0457-s001.pdf]

Supplementary Table 1. Multivariable Modified Poisson Regression Models of Factors Influencing Opposition to Guidelines for Provision of Sales Information for Prescription Drugs among Male Population.

Variables	aIRR (95%CI)	P-values
Interaction with MRs		
Rare interaction	1	
Moderate interaction	1.50 (1.19 - 1.89)	<0.001
Frequent interaction	1.47 (1.10 - 1.97)	0.010
Graduate year		
2010s	1	
2000s	0.84 (0.72 - 0.98)	0.028
1990s	0.71 (0.58 - 0.87)	<0.001
1980s or before	0.83 (0.70 - 0.99)	0.035
Types of affiliation		
National and public medical institutions	1	
Private and other medical institutions	1.16 (1.03 - 1.31)	0.014
Position		
No specific position	1	
Hospital director, hospital manager or clinic director	1.25 (1.07 - 1.48)	0.005
Head of Department/Professor	1.13 (0.97 - 1.32)	0.124
Decreased frequency of out-of-hospital events		
No	1	
Yes	1.36 (1.23 - 1.50)	<0.001
Content of the information provided by the MR became more superficial than the past		
No	1	
Yes	1.49 (1.35 - 1.65)	<0.001

CI: confidence interval; aIRR: adjusted incidence rate ratio; MR: medical representative.

Supplementary Table 2. Multivariable Modified Poisson Regression Models of Factors Influencing Opposition to Guidelines for Provision of Sales Information for Prescription Drugs among Female Population.

Variables	aIRR (95%CI)	P-values
Interaction with MRs		
Rare interaction	1	
Moderate interaction	1.57 (0.91 - 2.70)	0.102
Frequent interaction	2.67 (1.46 - 4.88)	0.001
Graduate year		
2010s	1	
2000s	0.97 (0.63 - 1.49)	0.880
1990s	0.49 (0.21 - 1.12)	0.090
1980s or before	0.72 (0.39 - 1.34)	0.303
Types of affiliation		
National and public medical institutions	1	
Private and other medical institutions	1.05 (0.73 - 1.53)	0.787
Position		
No specific position	1	
Hospital director, hospital manager or clinic director	0.77 (0.29 - 2.07)	0.606
Head of Department/Professor	1.58 (0.97 - 2.58)	0.067
Decreased frequency of out-of-hospital events		
No	1	
Yes	1.30 (0.88 - 1.91)	0.186
Content of the information provided by the MR became more superficial than the past		
No	1	
Yes	1.53 (0.999 - 2.34)	0.051

CI: confidence interval; aIRR: adjusted incidence rate ratio; MR: medical representative.

Supplementary Table 3: Characteristics of Respondents, Excluding the Fastest 10% to Complete the Survey.

Variable	Total (n = 1,080)	Frequent interaction (n=54)	Moderate interaction (n=858)	Rare interaction (n=168)
Female, n (%)	125 (11.6)	7 (13.0)	77 (9.0)	41 (24.4)
Age, n (%)				
＜40 years old	506 (46.9)	26 (48.2)	402 (46.9)	78 (46.4)
≧40 years old	574 (53.2)	28 (51.9)	456 (53.2)	98 (53.6)
Graduate year				
1950s or before	15 (1.4)	2 (3.7)	12 (1.4)	1 (0.6)
1960s	19 (1.8)	0 (0.0)	15 (1.8)	4 (2.4)
1970s	65 (6.0)	3 (5.6)	51 (5.9)	11 (6.6)
1980s	229 (21.2)	10 (18.5)	194 (22.6)	25 (14.9)
1990s	183 (16.9)	7 (13.0)	139 (16.2)	37 (22.0)
2000s	313 (29.0)	18 (33.3)	237 (27.6)	58 (34.5)
2010s	256 (23.7)	14 (25.9)	210 (24.5)	32 (19.1)
Type of affiliations, n (%)				
National/public university hospital	177 (16.4)	10 (18.5)	144 (16.8)	23 (13.7)
Private university hospital	66 (6.1)	9 (16.7)	54 (6.3)	3 (1.8)
National/public hospital	223 (20.7)	8 (14.8)	187 (21.8)	28 (16.7)
Private hospital	334 (30.9)	17 (31.5)	253 (29.5)	64 (38.1)
National/public clinic	15 (1.4)	1 (1.9)	12 (1.4)	2 (1.2)
Private clinic	252 (23.3)	9 (16.7)	205 (23.9)	38 (22.6)
Other	13 (1.2)	0 (0.0)	3 (0.4)	10 (6.0)
Position, n (%)				
Hospital director/manager	105 (9.7)	7 (13.0)	90 (10.5)	8 (4.8)
Clinic director	135 (12.5)	7 (13.0)	123 (14.3)	5 (3.0)
Department head/professor	240 (22.2)	18 (18.5)	198 (23.1)	32 (19.1)
No specific position	541 (50.1)	26 (48.2)	406 (47.3)	109 (64.9)
Other	59 (5.5)	4 (7.4)	41 (4.8)	14 (8.3)
Work status, n (%)				
Full-time	811 (75.1)	37 (68.5)	667 (77.7)	107 (63.7)
Full-time + part-time	179 (16.6)	10 (18.5)	140 (16.3)	29 (17.3)
Part-time only	90 (8.3)	7 (13.0)	51 (5.9)	32 (19.1)
Specialty, n (%)				
Internal Medicine	242 (22.4)	21 (38.9)	189 (22.0)	32 (19.1)
Respiratory Medicine	35 (3.2)	2 (3.7)	31 (3.6)	2 (1.2)
Gastroenterology	82 (7.6)	4 (7.4)	71 (8.3)	7 (4.2)
Cardiology	69 (6.4)	7 (13.0)	52 (6.1)	10 (6.0)
Pediatrics	84 (7.8)	0 (0.0)	74 (8.6)	10 (6.0)
Psychiatry	84 (7.8)	3 (5.6)	71 (8.3)	10 (6.0)
Psychosomatic Medicine	2 (0.2)	0 (0.0)	1 (0.1)	1 (0.6)
Allergology	0 (0.0)	0 (0.0)	0 (0.0)	0 (0.0)
Rheumatology	12 (1.1)	2 (3.7)	10 (1.2)	0 (0.0)
Surgery	64 (5.9)	3 (5.6)	53 (6.2)	8 (4.8)
Orthopedics	57 (5.3)	2 (3.7)	52 (6.1)	3 (1.8)
Plastic Surgery	11 (1.0)	0 (0.0)	5 (0.6)	6 (3.6)
Neurosurgery	24 (2.2)	0 (0.0)	22 (2.6)	2 (1.2)
Thoracic Surgery	6 (0.6)	0 (0.0)	2 (0.2)	4 (2.4)
Cardiovascular Surgery	10 (0.9)	0 (0.0)	7 (0.8)	3 (1.8)
Obstetrics and Gynecology	32 (3.0)	1 (1.9)	20 (2.3)	11 (6.6)
Obstetrics	3 (0.3)	0 (0.0)	3 (0.4)	0 (0.0)
Gynecology	3 (0.3)	0 (0.0)	1 (0.1)	2 (1.2)
Ophthalmology	21 (1.9)	1 (1.9)	17 (2.0)	3 (1.8)
Otolaryngology	33 (3.1)	0 (0.0)	29 (3.4)	4 (2.4)
Dermatology	36 (3.3)	1 (1.9)	31 (3.6)	4 (2.4)
Urology	18 (1.7)	0 (0.0)	17 (2.0)	1 (0.6)
Rehabilitation Medicine	7 (0.7)	0 (0.0)	3 (0.4)	4 (2.4)
Radiology	30 (2.8)	0 (0.0)	24 (2.8)	6 (3.6)
Anesthesiology	44 (4.1)	3 (5.6)	23 (2.7)	18 (10.7)
Other	71 (6.6)	4 (7.4)	50 (5.8)	17 (10.1)
Duration of individual meetings with MRs, n (%)				
10 minutes or less	684 (66.9)	28 (51.9)	565 (67.0)	91 (73.4)
11 – 20 minutes	261 (25.5)	21 (38.9)	218 (25.8)	22 (17.7)
21 – 30 minutes	62 (6.1)	4 (7.4)	51 (6.0)	7 (5.7)
31 – 60 minutes	15 (1.5)	1 (1.9)	10 (1.2)	4 (3.2)

MR: medical representative.

Supplementary Table 4: The Impact of Medical Representative Interaction Frequency on Physicians' Guideline Understanding, Implementation, and Perspectives, Excluding the Fastest 10% to Complete the Survey.

Variable	Total (N = 1080)	Frequent interaction (N=54)	Mild interaction (N=858)	Rare interaction (N=168)
Awareness and understanding about the GL (N, %)				
Not only be aware of the GL publication but also understand its contents	214 (19.8)	19 (35.2)	183 (21.3)	12 (7.1)
While being aware of the publication of the GL, not understand its contents	632 (58.5)	32 (59.3)	497 (57.9)	103 (61.3)
Be not aware of the publication of the GL	234 (21.7)	3 (5.6)	178 (20.8)	53 (31.6)
Changes after GL compliance (N, %)				
Decreased frequency of visits with MR	385 (35.7)	24 (44.4)	312 (36.4)	49 (29.2)
Decreased frequency of in-hospital briefings	361 (33.4)	28 (51.9)	291 (33.9)	42 (25.0)
Decreased frequency of out-of-hospital events	388 (35.9)	25 (46.3)	326 (38.0)	37 (22.0)
Content of the information provided by the MR became more superficial than the past	279 (25.8)	21 (38.9)	237 (27.6)	21 (12.5)
Publications of Guidelines and other regulations (N, %)				
Agree	64 (5.9)	3 (5.6)	46 (5.4)	15 (8.9)
Somewhat agree	224 (20.7)	9 (16.7)	170 (19.8)	45 (26.8)
Neutral	210 (19.4)	2 (3.7)	155 (18.1)	53 (31.6)
Somewhat disagree	314 (29.1)	16 (29.6)	266 (31.0)	32 (19.1)
Disagree	268 (24.8)	24 (44.4)	221 (25.8)	23 (13.7)

MR: medical representative; GL: Guideline.

Supplementary Table 5: Multivariable Modified Poisson Regression Models of Factors Influencing Opposition to Guidelines for Provision of Sales Information for Prescription Drugs, Excluding the Fastest 10% to Complete the Survey.

Variables	aIRR (95%CI)	P-values
Interaction with MRs		
Rare interaction	1	
Moderate interaction	1.47 (1.18 - 1.83)	0.001
Frequent interaction	1.77 (1.36 - 2.30)	<0.001
Graduate year		
2010s	1	
2000s	0.80 (0.68 - 0.93)	0.005
1990s	0.68 (0.56 - 0.84)	<0.001
1980s or before	0.83 (0.70 - 0.99)	0.041
Types of affiliation		
National and public medical institutions	1	
Private and other medical institutions	1.16 (1.03 - 1.31)	0.013
Position		
No specific position		
Hospital director, hospital manager or clinic director	1.22 (1.04 - 1.43)	0.016
Head of Department/Professor	1.15 (0.98 - 1.35)	0.083
Decreased frequency of out-of-hospital events		
No	1	
Yes	1.30 (1.18 - 1.45)	<0.001
Content of the information provided by the MR became more superficial than the past		
No	1	
Yes	1.49 (1.34 - 1.64)	<0.001

CI: confidence interval; aIRR: adjusted incidence rate ratio; MR: medical representative.

Supplementary Table 6. Analysis of Healthcare Professionals' Comments on Guidelines for Provision of Sales Information for Prescription Drugs, Excluding the Fastest 10% to Complete the Survey.

	Total	Agree	Neutral	Disagree
Agreement				
Focusing on money and collusion	38 (9.9)	30 (32.3)	2 (3.7)	6 (2.5)
Focusing on etiquette, burden, or discomfort	5 (1.3)	3 (3.2)	2 (3.7)	0 (0.0)
Focusing on patient benefits	4 (1.0)	3 (3.2)	1 (1.9)	0 (0.0)
Focusing on alternative information sources	12 (3.1)	5 (5.4)	2 (3.7)	5 (2.1)
Disagreement				
Tolerating financial transaction or gift-giving	87 (22.6)	8 (8.6)	4 (7.4)	75 (31.5)
Focusing on information gathering or patient disadvantages	87 (22.6)	7 (7.5)	8 (14.8)	72 (30.3)
Comments difficult to be categorized	152 (39.5)	37 (39.8)	35 (64.8)	80 (33.6)
Missing	695	195	156	344
